# Characterization of Fibrillar Collagens and Extracellular Matrix of Glandular Benign Prostatic Hyperplasia Nodules

**DOI:** 10.1371/journal.pone.0109102

**Published:** 2014-10-02

**Authors:** Tyler M. Bauman, Tristan M. Nicholson, Lisa L. Abler, Kevin W. Eliceiri, Wei Huang, Chad M. Vezina, William A. Ricke

**Affiliations:** 1 Department of Urology, University of Wisconsin School of Medicine and Public Health, Madison, WI, United States of America; 2 Department of Comparative Biosciences, University of Wisconsin School of Veterinary Medicine, Madison, WI, United States of America; 3 Laboratory for Optical and Computational Instrumentation, University of Wisconsin Graduate School, Madison, WI, United States of America; 4 Department of Pathology and Laboratory Medicine, University of Wisconsin School of Medicine and Public Health, Madison, WI, United States of America; 5 Carbone Cancer Center, University of Wisconsin School of Medicine and Public Health, Madison, WI, United States of America; National Cancer Institute, National Institutes of Health, United States of America

## Abstract

**Objective:**

Recent studies have associated lower urinary tract symptoms (LUTS) in men with prostatic fibrosis, but a definitive link between collagen deposition and LUTS has yet to be demonstrated. The objective of this study was to evaluate ECM and collagen content within normal glandular prostate tissue and glandular BPH, and to evaluate the association of clinical parameters of LUTS with collagen content.

**Methods:**

Fibrillar collagen and ECM content was assessed in normal prostate (48 patients) and glandular BPH nodules (24 patients) using Masson's trichrome stain and Picrosirius red stain. Second harmonic generation (SHG) imaging was used to evaluate collagen content. Additional BPH tissues (n = 47) were stained with Picrosirius red and the association between clinical parameters of BPH/LUTS and collagen content was assessed.

**Results:**

ECM was similar in normal prostate and BPH (p = 0.44). Total collagen content between normal prostate and glandular BPH was similar (p = 0.27), but a significant increase in thicker collagen bundles was observed in BPH (p = 0.045). Using SHG imaging, collagen content in BPH (mean intensity = 62.52; SEM = 2.74) was significantly higher than in normal prostate (51.77±3.49; p = 0.02). Total collagen content was not associated with treatment with finasteride (p = 0.47) or α-blockers (p = 0.52), pre-TURP AUA symptom index (p = 0.90), prostate-specific antigen (p = 0.86), post-void residual (PVR; p = 0.32), prostate size (p = 0.21), or post-TURP PVR (p = 0.51). Collagen content was not associated with patient age in patients with BPH, however as men aged normal prostatic tissue had a decreased proportion of thick collagen bundles.

**Conclusions:**

The proportion of larger bundles of collagen, but not total collagen, is increased in BPH nodules, suggesting that these large fibers may play a role in BPH/LUTS. Total collagen content is independent of clinical parameters of BPH and LUTS. If fibrosis and overall ECM deposition are associated with BPH/LUTS, this relationship likely exists in regions of the prostate other than glandular hyperplasia.

## Introduction

Lower urinary tract symptoms (LUTS) are a major medical problem with an estimated prevalence of 20% in men over 20 [1] and 70% in men over 80 [2]. LUTS comprise a spectrum of symptoms including nocturia, weak stream, urgency, slow stream, and a sensation of incomplete emptying, among others [3]. The most common cause of LUTS in older men is benign prostatic hyperplasia (BPH), which histologically consists of epithelial and stromal nodules in the transition zone of the prostate. BPH can lead to acute urinary retention, recurrent urinary tract infections, hematuria, and renal insufficiency [4] and represents a serious disease that affects a large proportion of the male population.

A typical treatment regimen for symptomatic BPH includes α-adrenergic antagonists (α-blockers) to relax smooth muscle and 5α-reductase inhibitors (5α-RIs) such as finasteride or dutasteride to block the conversion of testosterone to the more potent androgen dihydrotestosterone [4]. Despite medical therapy, many patients undergo surgical intervention if symptoms persist. The gold standard surgical treatment for BPH is transurethral resection of the prostate (TURP). While there are multiple non-prostatic explanations for LUTS [5], one proposed reason for treatment failure with α-blockers or 5α-RIs is collagen deposition within regions of the prostate as a result of fibrosis [6], [7].

A previous study involving mechanical testing of prostate tissue rigidity demonstrated an association between LUTS and tissue stiffness [6]. Additionally, quantification of collagen in the extracellular matrix (ECM) through Masson's trichrome stain has associated ECM collagen content with tissue rigidity, implying that collagen deposition and hence fibrosis may be a previously unidentified variable contributing to lower urinary tract symptomology [6]. While it is becoming increasing clear that fibrosis in the prostate is an important area of study, there is currently a void of knowledge on basic collagen expression patterns in the prostate. The purpose of the present study was to characterize collagen and ECM content in glandular human prostate tissues. We hypothesized that collagen content and fiber thickness would be increased in glandular BPH nodules, and that collagen content in BPH nodules would predict symptoms as other studies have previously demonstrated [6], [8].

## Methods

The University of Wisconsin Institutional Review Board (IRB) (2012-1033, 2012-0508) approved retrospective review of patient information and demographics included in this study and waived the need for written informed consent from patients. Tissues were obtained from a pathology archive and were used for diagnostic purposes, so patient consent was not deemed necessary. Patient identifying information was anonymized and de-identified prior to analysis.

### Tissue microarray samples

A tissue microarray (TMA) of human prostate tissues was used in this study and has been previously described [9], [10]. Benign human prostate tissue was obtained from prostatectomy specimens from patients who were not treated with neoadjuvant hormonal therapies (96 cores, in duplicate, from 48 patients). BPH tissue was acquired from patients undergoing transurethral resection of the prostate (48 cores, in duplicate, from 24 patients). Surgical indications for BPH patients include a history of LUTS and failure of medical therapy. Each TMA core was 0.6 mm in diameter and arranged 0.2 mm apart both vertically and horizontally using a Manual Tissue Arrayer (Beecher Instruments, Sun Prairie, WI; Model MTA-1).

### Transurethral resection of the prostate BPH samples

After Institutional Review Board (IRB) approval (2012-1033, 2012-0508), patients undergoing transurethral resection of the prostate for treatment of BPH at the University of Wisconsin Hospital from 2004 to 2010 were identified using an institutional database. Patients included in the study were randomly selected and on initial chart review, patients with an ambiguous history of 5α-RI or α-blocker treatment for BPH were excluded. TURP specimens were fixed in 10% neutral buffered formalin and embedded in paraffin. 5-µm thick sections on positively charged microscope slides were acquired from the University of Wisconsin Pathology archive. This patient population has been further described previously [11]. An overview of experimental design is highlighted in **Figure S1**.

### Masson's trichrome staining

5-µm sections of formalin-fixed, paraffin-embedded (FFPE) tissues were de-paraffinized in xylene (3 washes for 3 min each) and hydrated in graded ethanol to distilled water. Slides were stained with Masson's trichrome as previously described [12], followed by dehydration in graded ethanol to xylene. Glass coverslips were applied using a resinous mounting medium (Thermo Scientific, Pittsburgh, PA).

### Picrosirius red staining

5-µm thick formalin-fixed, paraffin-embedded (FFPE) sections were briefly de-paraffinized at room temperature in four washes of xylene (3 min each). Sections were hydrated in graded ethanols (100%, 100%, 95%, 95%, 70%; all 1 min) to distilled water. Nuclei were stained using immersion in Weigert's iron hematoxylin (Sigma-Aldrich, St. Louis, MO) for 8 mins, followed by washing in running tap water for 10 mins. Sections were stained at room temperature in a solution of 0.1% Sirius red F3BA in saturated aqueous picric acid (Sigma-Aldrich) for one hour, as described in other studies [13], [14]. Subsequently, sections were rinsed in two washes of a 0.5% acetic acid in distilled water solution (5 min each). Ascending concentrations of ethanol were used for dehydration (70%, 95%, 95%, 100%, 100%; 4 quick dips each) and sections were cleared in three washes of xylene (4 quick dips). Sections were covered with resinous mounting medium and glass cover slips.

### Image acquisition and analysis

Images of normal prostate and glandular BPH from the TMA stained with Masson's trichrome were acquired with a 20x objective lens (N.A. = 0.50, Nikon Instruments, Melville, NY) on an 80i microscope (Nikon) using the DS-Fi2 camera (Nikon) with NIS Elements (Nikon). Blue coloration, indicative of ECM, was separated for all cores by manual thresholding of hue (121-179), saturation (20-255), and brightness (10-255) values in ImageJ [15], and ECM content was quantified as mean blue intensity per tissue area. ECM content was compared between normal prostate and BPH using a two-tailed Student's t-test.

Images of Picrosirius red staining were acquired for each TMA core of interest using a 20x objective lens (N.A. = 0.50, Nikon) on the DS-Fi2 camera (Nikon) in NIS Elements (Nikon) using the Nikon DS-U3 controller (Nikon). For full slide TURP specimens, three representative acinar lobules were identified and imaged for each patient using the 10x objective lens (N.A. = 0.45, Nikon), and triplicate averages were used for analysis. Images were acquired using both brightfield microscopy and a circular polarizer filter, as birefringence under polarized light is highly specific for collagen [13], [14].

Quantification of polarized light images was conducted similar to previously established protocols [13] (**Figure S2**). Background coloration was removed using ImageJ [15] and the Colour Corrector plugin. ImageJ macros for the quantification of individual colors of birefringence were developed using the following hue (H), saturation (S), and brightness (B) ranges: red (H 1-13, S 10-255, B 20-255), orange (H 14-25, S 10-255, B 20-255), yellow (H 26-52, S 10-255, B 20-255), and green (H 53-110, S 10-255, B 20-255), a slight modification of hue range values from previously described methods [13]. Total collagen content was defined as the proportion of positive pixels within the birefringent bin ranges compared to total pixels in the region of interest (ROI) for each image. ROI sizes were manually calculated using Photoshop CS6 (Adobe Systems, San Jose, CA) by subtracting empty and glandular space from the total amount of pixels in each image. Proportion of colors within birefringent tissue was normalized and compared between groups. Student's t-test was used for statistical analysis using Graphpad Prism (Graphpad Software, San Diego, CA), with a two-sided p-value of <0.05 being considered significant in all analyses.

### Second harmonic generation imaging

A custom multiphoton workstation [16]–[20] at the Laboratory for Optical and Computational Instrumentation (LOCI) was used to image tissue slides with second harmonic generation (SHG) imaging using a TE300 inverted microscope (Nikon, Tokyo, Japan) equipped with a CFI Plan Apo x40 (N.A = 1.15; Nikon) objective lens and a mode-locked Ti: Sapphire laser (Mai Tai Deepsee; Spectra Physics, Mountain View, CA). The excitation wavelength was tuned to 890 nm; a 445 nm±25 nm narrow bandpass emission filter (Semrock) was used to detect the SHG signal of collagen in the backscattered mode using a H7422P GaAsP photon counting PMT (Hamamatsu Photonics, Hamamatsu City, Japan). Images of 1024×1024 pixels were acquired using WiscScan (LOCI, University of Wisconsin, Madison). Collagen content was compared between glandular BPH (n = 11) and normal prostate tissue (n = 10) using randomly selected cores from the TMA [21], [22]. A total of 12–15 optical sections were acquired per core, and image stacks were flattened using Fiji [23] software to create maximum intensity Z projections. Collagen content was quantified as mean gray intensity in ImageJ and the Student's t-test was used for statistical analysis, with a two-sided p-value of <0.05 being considered significant.

## Results

### Extracellular matrix composition in prostate tissues

**Figure 1:**
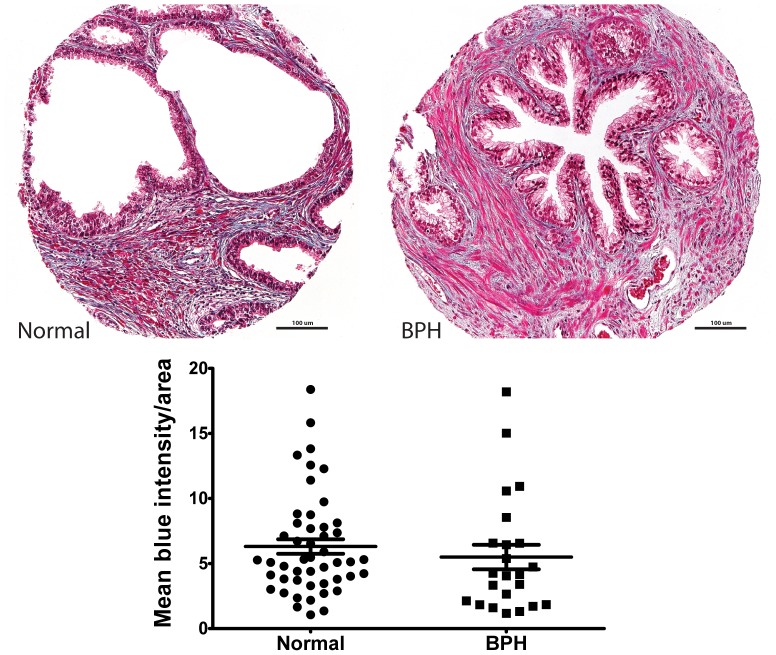
Extracellular matrix content is similar in normal prostate tissue and glandular BPH. The tissue microarray (TMA) was stained with Masson's trichrome for detection of cellular and extracellular matrix (ECM) contents. Blue coloration, indicative of ECM, was separated for all cores by manual thresholding of hue (121–179), saturation (20–255), and brightness (10–255) values in ImageJ, and ECM content was quantified as mean blue intensity per tissue area. ECM composition was similar in normal prostate (mean = 6.308; SEM = 0.560) and glandular BPH (5.496±0.940; p = 0.44).

To evaluate the ECM content, we first stained prostate tissues for Masson's trichrome and quantified the intensity of blue (representing extracellular matrix) within prostate tissue area. Blue intensity was not significantly different between normal prostate tissue (mean = 6.308; SEM = 0.560) and glandular BPH (5.496±0.940; p = 0.44; **Figure 1**), indicating that ECM content is similar in normal prostate tissues and BPH.

### Evaluation of collagen content in normal prostate and BPH by Picrosirius red staining

**Figure 2:**
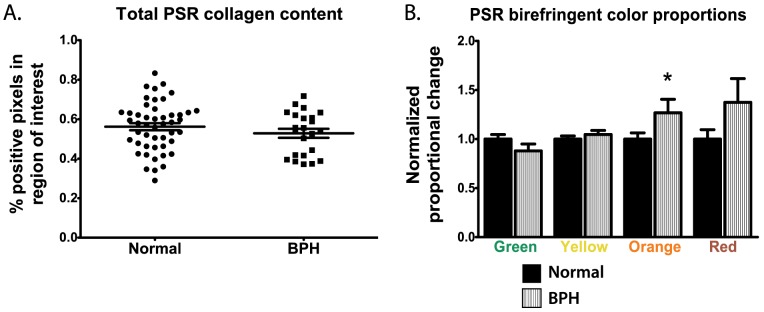
Analysis of fibrillar collagen content in normal prostate tissues and glandular BPH with Picrosirius red staining. Total collagen content was defined as the sum of positive birefringent pixels divided by the total size of the region of interest. Total collagen content was not significantly different in normal prostate tissue (mean = 56.2%; SEM = 1.8) and glandular BPH (52.9±2.3; p = 0.27; **A**). The normalized proportion of green (p = 0.15), yellow (p = 0.39), and red (p = 0.09) birefringent collagen bundles were not significantly different between normal prostate tissues and BPH (**B**). The proportion of orange bundles was significantly higher in glandular BPH (normalized mean = 1.268; SEM = 0.138) than normal prostate (1.000±0.063; p = 0.045).

To investigate fibrillar collagen levels in normal prostate and glandular BPH, we then stained the TMA with Picrosirius red and acquired images under circularly polarized light. Total birefringence was not significantly different between normal prostate (mean = 56.2%; SEM = 1.8) and glandular BPH (52.9±2.3; p = 0.27; **Figure 2**). In further analysis, the normalized proportion of green (normalized mean±SEM = 0.879±0.070 vs. 1.000±0.046; p = 0.15) and yellow (1.047±0.041 vs. 1.000±0.032; p = 0.39) birefringence was not significantly different between normal prostate and BPH. The proportion of orange birefringence, corresponding to thicker collagen bundles, was significantly higher in BPH (1.268±0.138 vs. 1.000±0.063; p = 0.045). There was no significant difference in red birefringence, corresponding to the thickest collagen bundles (1.374±0.242 vs. 1.000±0.096; p = 0.09).

### Collagen content in TMA cores using second harmonic generation imaging

**Figure 3:**
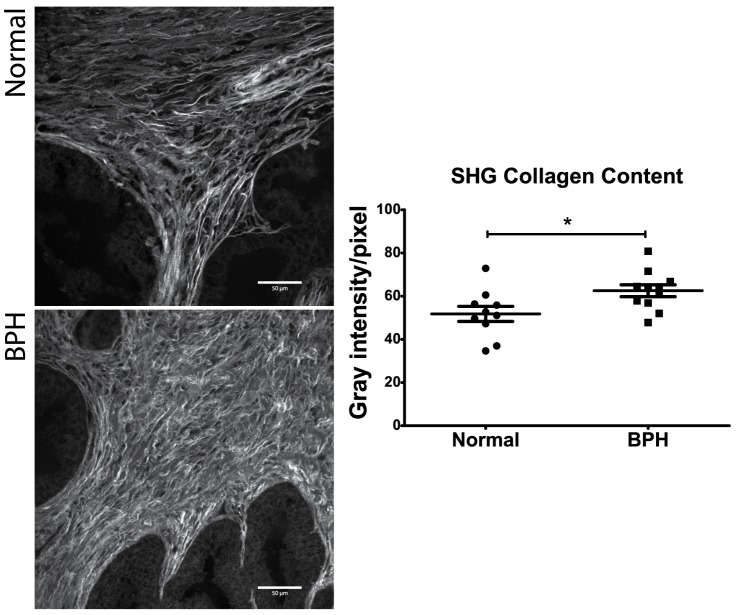
Evaluation of collagen content in normal prostate and glandular benign prostatic hyperplasia (BPH) with second harmonic generation (SHG) imaging. Using SHG imaging, a total of 12–15 optical sections were acquired (40x objective) per core from a subset of normal prostate tissue (n = 10) and BPH (n = 11). Optical sections were stacked and flattened to create maximum intensity Z-projections using Fiji software. Collagen content was quantified as the mean gray intensity within the region of interest. Using SHG imaging, a significant increase in collagen content was observed in BPH (mean = 62.52, SEM = 2.738) compared to normal prostate tissue (51.77±3.492; p = 0.02).

To further investigate levels of thicker collagen bundles in TMA samples, the collagen content in normal prostate and glandular BPH was assessed using second harmonic generation imaging, which has previously been shown to primarily detect thicker type I collagen [21]. Collagen content was significantly higher in BPH nodules (mean = 62.52, SEM = 2.74) than normal prostate (51.77±3.49; p = 0.02; **Figure 3**).

### Association of collagen content and clinical parameters of BPH

We then investigated the association of collagen content with different clinical parameters of BPH and LUTS by staining an additional set of TURP specimens from 47 patients with Picrosirius red. Available clinical parameters for analysis included pre-TURP medical treatment information (all patients), pre-TURP PSA (n = 34; median = 4.05 ng/ml; IQR 2.10–7.03), pre-TURP prostate size (n = 15, median = 35 g; IQR 28.8-40), pre-TURP AUA symptom index (AUASI; n = 15; median = 21; IQR 19.5–27), pre-TURP post-void residual (PVR; n = 19; median = 164.0 ml; IQR 117.5–479.5), and post-TURP PVR (n = 24; median = 80 ml; IQR 24–252; **Table 1**).

**Table 1:** Relationship of total collagen content with clinical characteristics of patient population from analysis of TURP specimens.

Characteristic	No. available (%)	Median [IQR]	Slope ± SEM	R^2^	p-value
Pre-TURP prostate size (g)	15 (31.9)	35.0 [28.8–40.0]	66.52±50.73	0.117	0.21
Pre-TURP AUA symptom index	15 (31.9)	21.0 [19.5–27.0]	2.731±22.06	0.001	0.90
Pre-TURP post-void residual (ml)	19 (40.4)	164 [117.5–479.5]	471.2±460.6	0.058	0.32
Post-TURP post-void residual (ml)	24 (51.1)	79.5 [24.0–251.5]	0.3380±0.5065	0.018	0.51
Pre-TURP PSA (ng/ml)	34 (72.3)	4.05 [2.10–7.03]	−1.019±5.627	0.001	0.86

**Abbreviations**: transurethral resection of the prostate (TURP), American Urological Association (AUA), interquartile range (IQR), prostate-specific antigen (PSA), number (No.).

**Figure 4:**
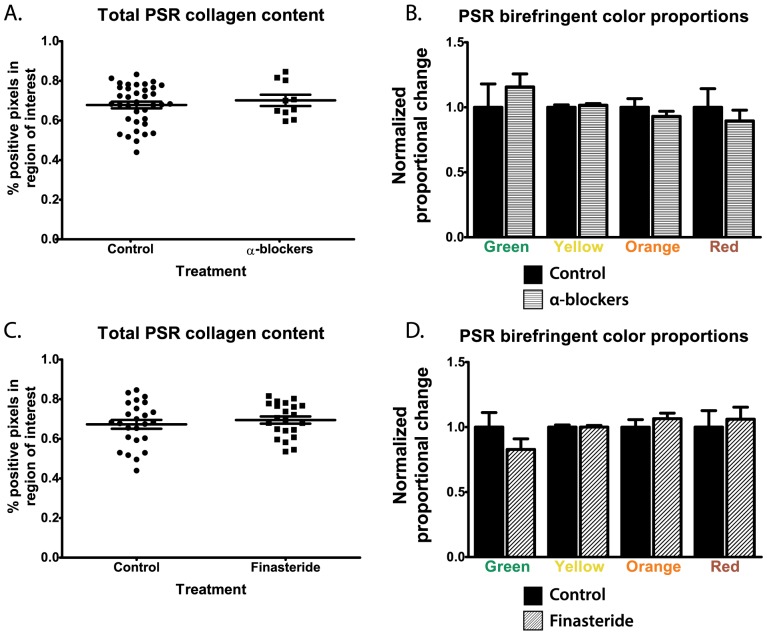
Association of Picrosirius red fibrillar collagen content in glandular BPH specimens with pre-TURP treatment with α-blockers and finasteride. A total of 47 patients undergoing transurethral resection of the prostate (TURP) for treatment of BPH at the University of Wisconsin Hospital were randomly selected for inclusion in this study. TURP samples were stained for Picrosirius red, and 3 representative acinar lobules were imaged under polarized light. Staining was quantified as total collagen content and proportion of colors within birefringent tissue, and triplicate images for each patient were averaged. Treatment with α-blockers (n = 37 patients) had no significant effect on total collagen content in glandular BPH nodules (p = 0.52; **A**). No changes were observed in the proportion of green (normalized mean = 1.157±0.010 vs. 1.000±0.180; p = 0.47), yellow (1.016±0.013 vs. 1.000±0.019; p = 0.55), orange (0.930±0.039 vs. 1.000±0.067; p = 0.40), or red (0.896±0.082 vs. 1.000±0.143; p = 0.55) birefringent tissue (**B**). Similarly, treatment with finasteride (n = 22 patients) had no effect on total collagen content within the tissues (p = 0.47; **C**). No significant changes in the proportion of green (0.828±0.082 vs. 1.000±0.112; p = 0.23), yellow (1.000±0.013 vs. 1.000±0.017; p = 1.00), orange (1.065±0.042 vs. 1.000±0.058; p = 0.38), or red (1.061±0.092 vs. 1.000±0.127; p = 0.71) collagen bundles were observed (**D**).

We hypothesized that the medical management of BPH and LUTS with 5α-RIs and α-blockers may be leading to an increase in fibrosis within the prostate. Patients treated with α-blockers (n = 37) had no significant difference in total collagen content (p = 0.52) or in Picrosirius red birefringent color distribution compared to patients not treated with α-blockers (n = 10; **Figure 4**). Of the total 47 patients, 22 received treatment with finasteride prior to TURP (median treatment length = 7.5 months; IQR 3.5–30) for alleviation of BPH symptoms. The other 25 patients had no history of 5α-RI treatment. Specimens from patients treated with finasteride had no significant difference in total collagen content compared to patients not on 5α-RIs (p = 0.47), and no difference in the distribution of bundle size within the birefringent tissue was observed.

**Figure 5:**
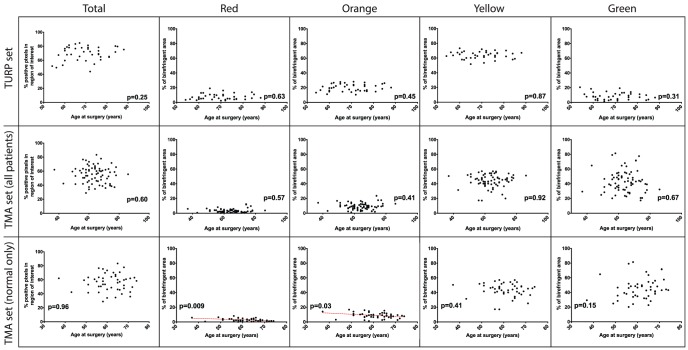
Association of patient age with Picrosirius red collagen content in prostate tissues. The association of Picrosirius red collagen content and patient age at time of surgery was investigated in cohorts of BPH only tissues (TURP set), a combination of BPH and normal prostate tissue (TMA set [all patients]), and a subset of only normal prostate tissue (TMA set [normal only]). Total collagen content and the distribution of birefringent colors were not associated with age in the TURP set or combination TMA set (all patients) of tissues (p>0.05). Total collagen content was not associated with age in the TMA subset of normal only patients (p = 0.96), but a significant negative correlation between age and birefringent color proportions was observed for red (p = 0.009) and orange (p = 0.03) birefringence. No association was found between age and yellow (p = 0.41) or green (p = 0.15) birefringence in the normal only subset of prostate tissues.

We then investigated the association between total collagen content, as measured by Picrosirius red birefringence, with other clinical parameters of our patient population. We found no significant association between total collagen content and pre-TURP PSA (p = 0.86), pre-TURP AUASI (p = 0.90), pre-TURP PVR (p = 0.32), post-TURP PVR (p = 0.85), or pre-TURP prostate size (p = 0.21; **Table 1**). Furthermore, pre-TURP AUASI was not associated with the proportion of thicker red (p = 0.67) or orange (p = 0.95) birefringence (data not shown). Patient age at time of surgery was not associated with total Picrosirius red birefringence in BPH samples (p>0.05) or in a combination of BPH and normal prostate samples (p>0.05; **Figure 5**). In a sub-analysis of only normal prostate samples, red and orange birefringence were negatively correlated with patient age (p<0.05).

## Discussion

BPH causes significant morbidity and decreases the quality of life of older men [2], and represents a substantial financial burden to society [4], [24]. Current medical therapies relax smooth muscle within the transition zone of the prostate (α-blockers) and block the conversion of testosterone to DHT (5α-RIs). These treatment options have largely been effective in decreasing the risk of urinary retention, lowering the rate of invasive intervention, and reducing LUTS, particularly when used in combination [25], [26]. Despite the general effectiveness of current treatment options, a substantial proportion of patients are unresponsive to medical therapy and undergo costly surgical intervention [24]. Furthermore, many patients progress in disease after initially responding well to these therapies. Discovering why some patients are unresponsive to α-blockers and 5α-RIs will allow for the introduction of alternative treatment options, thus reducing the need for TURP or laser ablation and the costs and risks associated with surgery [24], [27].

One theory on why certain patients are unresponsive to treatment for LUTS is the presence of increased fibrosis within the prostate. Collagen fibers, which are one primary aspect of fibrosis, play an important role in maintaining structural integrity, but excess accumulation has been shown to be detrimental in many other disease states [28]–[31]. The role of fibrosis and collagen deposition has recently gained attention in BPH. Previously, mechanical testing of transition zone slices from prostatectomy specimens mounts showed an association between AUA symptom score and tissue rigidity [6]. Quantification of Masson's trichrome staining in these specimens demonstrated an association between tissue rigidity and ECM content, suggesting that deposition of collagen into the ECM might be contributing to symptomology in some patients. One additional prospective study investigated periurethral fibrosis using Verhoeff-van Gieson (VVG) staining of prostatectomy specimens [8]. In this study, periurethral biopsy cores were taken from prostatectomy specimens, and VVG staining was used to assess elastin and collagen content. The authors concluded that collagen content was positively correlated with International Prostatic Symptoms Score (IPSS) and extent of inflammation, supporting previous findings [6].

While it is becoming increasingly clear that prostatic fibrosis and fibrillar collagen deposition is potentially associated with LUTS in some patients, studies to this point have been limited in both tissue type and staining specificity. With the recent decline in TURP and increase in ablation approaches for the surgical management of BPH [32], acquisition of benign prostate tissue has become increasingly difficult; therefore, previous studies have often used prostatectomy specimens for the evaluation of collagen content [6], [8]. Even when controlling for prostate zone where malignancy is occurring, the links between prostate cancer and inflammation and fibrosis [33], [34] are concerning. Furthermore, stains such as Verhoeff-van Gieson and Masson's trichrome, which are not specific to fibrillar collagen, have been used rather than Picrosirius red, the latter of which has been shown to be highly specific for fibrillar collagens when imaged under circularly polarized light [13], [14]. No studies to date have directly compared collagen content in normal prostate and glandular BPH acquired from TURP using a multi-technique approach.

In the present study, we did not detect differences in ECM content between normal prostate and glandular BPH using Masson's trichrome staining. We also did not find difference in overall collagen levels between normal prostate tissue and glandular BPH nodules by quantification of Picrosirus red staining viewed under circularly polarized light. We did, however, observe a shift towards thicker collagen fibers in BPH nodules when individual birefringent colors were quantified. To further investigate this, we used second harmonic generation imaging on a subset of TMA cores from both groups. Since second harmonic generation imaging is most specific for thick, type I collagen [21], we confirmed our hypothesis that we would observe an increase in collagen content using this method.

Biological implications of these findings are both important and controversial. The shift towards thicker collagen fibers in glandular BPH nodules may add sufficient rigidity to the periurethral region to impact urinary function, but this is unlikely for a multitude of reasons. Collagen is linked to tensile strength rather than compressive strength [35]. Furthermore, in this study, overall ECM and fibrillar collagen levels were similar in normal prostate and glandular BPH, if not trending downward. While the increase in thicker collagen fibers may play an indirect role in BPH progression through mechanisms such as cell signaling [36], the likelihood that these small changes in collagen bundle thickness within BPH nodules are directly responsible for LUTS through physical mechanisms is low. Further studies are needed to determine if changes in collagen isoforms and other pro-fibrotic factors are associated with BPH/LUTS.

We then sought to identify the role of fibrosis in patients with LUTS secondary to BPH using a different set of TURP tissues. We hypothesized that fibrosis may be linked to only a small subset of patients undergoing TURP, and that medical management of BPH with 5α-RIs and α-blockers might be a contributing factor. In this set of patients, we previously demonstrated that treatment with finasteride results in basal cell hyperplasia [11], which is one hallmark of increased local estrogen levels [37]. Interestingly, higher estrogen levels can also result in increased collagen deposition [38], suggesting that estrogen-stimulated deposition of collagen by fibroblasts, myofibroblasts, and fibrocytes through 5α-RI treatment may be contributing to remaining LUTS. However, in this study, we found no association between treatment with finasteride and changes in collagen levels. Treatment with α-blockers was also not associated with fibrillar collagen levels in BPH nodules. We then aimed to investigate whether levels of collagen within glandular BPH tissues were associated with different clinical parameters of BPH such as pre-TURP post-void residual, prostate size, PSA level, and AUA symptom index. Despite our low sample size, our results indicate that levels of fibrillar collagen within BPH nodules are largely independent of these parameters.

Finally, using both sets of prostate tissues, we investigated the association between patient age at time of surgery and prostatic collagen levels. Collagen content and bundle thickness proportions did not change with age in our analyses involving BPH specimens. When we analyzed a subset of only normal prostate samples, we found no change in total collagen content but a significant decrease in the proportion of thicker collagen bundles as patients aged. These results align with previous animal studies on prostate collagen content over time [39] and support the idea that changes in prostatic collagen content are largely age-independent. Furthermore, if collagen deposition and fibrosis are associated with BPH/LUTS then these factors may be specific to disease progression and not simply due to aging.

Limitations of this study include original tissue collection for the TMA. Normal prostate tissue included on the TMA was acquired from patients undergoing prostatectomy for surgical management of prostate cancer. A limitation of our cohort of normal prostate tissues is that the exact zone or region in which tissue was taken is unknown. Each sample was taken from either the transition or peripheral zone, though the exact location was not noted at the time of tissue harvest. Therefore, it is probable that some normal prostate tissues were not from the transition zone, potentially making a direct comparison with BPH difficult. Additionally, while efforts were made to eliminate cores that appeared to be sampled from pure stromal BPH nodules, some glandular BPH tissue from our analysis was most likely sampled from mixed glandular/stromal nodules, rather than pure glandular BPH. While our results indicate that there is no difference in collagen content and ECM content between BPH and normal prostate tissue, it is possible that these confounding variables may be playing a role.

In all, our data suggest that while fibrosis may be an important contributing factor in LUTS in some patients, collagen deposition specifically within glandular BPH nodules is likely unrelated to LUTS. If fibrosis is indeed occurring and is associated with LUTS, it is likely occurring in regions of the prostate other than within glandular tissue. With the advent of new animal models for BPH/LUTS and a reassessment of stromal nodules and periurethral human tissues, elucidation of the role of collagen deposition/fibrosis is likely.

## Conclusions

Overall collagen levels are similar in normal prostate and glandular BPH nodules, but the proportion of thicker collagen bundles is elevated in glandular BPH nodules. Collagen levels, as described herein, are not associated with clinical parameters of BPH and LUTS, suggesting that fibrosis specifically within glandular nodules of BPH tissue is not a contributing factor in LUTS.

## Supporting Information

Figure S1 **Experimental design for evaluation of fibrillar collagen content and extracellular matrix (ECM) content in prostate tissues.** A tissue microarray (TMA) containing tumor-adjacent normal prostate tissue (96 cores, in duplicate, from 48 patients) and glandular BPH (48 cores, in duplicate, from 24 patients) was stained with Masson's trichrome (MTC) and Picrosirius red (PSR), and second harmonic generation (SHG) imaging was also used to identify collagen in a randomly sampled subset of TMA patients (n = 10 [normal prostate]; n = 11 [BPH]). ECM content and fibrillar collagen content was quantified and compared between normal prostate tissue and BPH. An additional set of full slide specimens acquired from transurethral resection of the prostate (TURP; n = 47) were stained with Picrosirius red, and relevant clinical information for these patients was collected using retrospective chart review. The association of collagen content and clinical parameters of BPH and LUTS was investigated.
(EPS)

Figure S2 **Separation and quantification of birefringence in Picrosirius red stained prostate tissues.** Prostate tissues were stained with Picrosirius red and imaged under brightfield (**A**) and circular polarized light (**B**) microscopy. Individual colors of birefringence were separated using thresholding of hue, saturation, and brightness values (**C**). ImageJ was used to quantify total birefringent area in the region of interest and the proportion of individual birefringent colors (**D**) comprising that area.
(EPS)
